# Distinct arsenic metabolites following seaweed consumption in humans

**DOI:** 10.1038/s41598-017-03883-7

**Published:** 2017-06-20

**Authors:** Vivien F. Taylor, Zhigang Li, Vicki Sayarath, Thomas J. Palys, Kevin R. Morse, Rachel A. Scholz-Bright, Margaret R. Karagas

**Affiliations:** 1 Department of Earth Science, 6105 Sherman Fairchild Hall, Dartmouth College, Hanover, NH 03755 USA; 2 Department of Epidemiology, Geisel School of Medicine, 1 Medical Center Drive 7927 Rubin Building, Lebanon, NH 03756 USA

## Abstract

Seaweeds contain arsenic primarily in the form of arsenosugars, which can be metabolized to a wide range of arsenic compounds. To characterize human exposure to arsenic from seaweed consumption, we determined concentrations of arsenic species in locally available seaweeds, and assessed urinary arsenic compounds in an experimental feeding study. A total of 11 volunteers consumed 10 g per day of three types of seaweeds (nori, kombu, and wakame) for three days each, while abstaining from rice and seafood following a three-day washout period. Urinary arsenosugars and their metabolites (including dimethyl arsenate (DMA), thio-dimethylarsinoylethanol (thio-DMAE), thio-dimethylarsinoylacetate (thio-DMAA), and thio-DMA) were measured in spot urine samples prior to seaweed consumption, and in 24-hour urine samples while consuming seaweed. Commercial products made from whole seaweed had substantial concentrations of arsenic (12–84 µg/g), dominated by arsenosugars. Intact arsenosugars along with DMA, thio-DMAA, thio-DMAE all increased in urine after ingesting each type of seaweed, and varied between seaweed types and between individuals. Only trace levels of the known toxic metabolite, thio-DMA, were observed, across individuals. Thio-DMAE and thio-DMAA are unique products of arsenosugar breakdown, thus assessment of these compounds may help to identify dietary intake of arsenic from seaweed from other exposure pathways.

## Introduction

Commonly consumed seaweeds are known to contain high levels of arsenic, primarily in organic forms^1^. While inorganic arsenic (iAs) is an established human carcinogen^2^, less is known about organic arsenic species, such as arsenosugars found in seaweeds. Arsenosugars can be metabolized to a variety of compounds^3,4^, with at least one metabolite, thio-dimethyl arsenic (thio-DMA), showing cytotoxicity in bladder and lung cells^5–9^. The presence of arsenic in seaweed products, and its ability to form potentially toxic species has raised concern about possible human health impacts of seaweed consumption.

Populations throughout the world consume seaweed, particularly in Asia where seaweed can be a dietary staple. In Japan, the population consumes an estimated ~1 mg/day of arsenosugar, primarily due to seaweed consumption^10^. In North America and Europe, seaweed consumption is less common, and like other seafood, intake is likely high among some ethnic and regional sub-populations^11^. Seaweed products have become widely commercially available^12^, and include many seaweed types, with nori (red algae), kombu and wakame (both brown algae) being the most common^13^.

Despite the availability of seaweed products and concern about arsenic exposure from food sources^14,15^, there is a lack of information evaluating this source of arsenic. Understanding exposure to seaweed arsenic requires assessment of the levels of arsenic in common seaweeds, the extent of absorption following consumption, and the levels of arsenic compounds formed in the body. While arsenic levels and speciation have been assessed in seaweeds from various harvesting regions^16–18^, few studies have evaluated edible seaweed products marketed to a particular population, and most of these focus on iAs^12,19–21^. Indeed, early feeding studies of seaweed reported unknown arsenic compounds in human urine^22,23^. Following intake of synthetic arsenosugar, more than 10 other metabolite species have been characterized in urine^3,4^, including the compounds thio-dimethylarsinoylethanol (thio-DMAE) and thio-dimethylarsinoylacetate (thio-DMAA), which were the most abundant metabolites after DMA (Table 1S Supplemental). As yet, these compounds have not been measured following consumption of seaweed. Only one prior study from China has linked consumption of seaweed to urinary levels of multiple arsenic species across individuals^24^, but the seaweed used was not specified or characterized quantitatively. The study also relied on spot urine samples (rather than 24 h), and the laboratory lacked analytical standards and the speciation reported was not in line with other studies. Complete evaluation of arsenic species from real food samples is important to understanding human exposure and interpreting arsenic sources from urinary biomarkers. In this work, we conducted a market basket assessment and performed an experimental feeding study to gain a more complete understanding of exposure to arsenic in seaweed products typically marketed in the USA. To accomplish this, we used a repeated consumption design with three different seaweed types, where 24 h urine samples were collected throughout the feeding period, and extensively characterized the arsenic compounds excreted in urine, including the arsenosugar metabolites thio-DMAE and thio-DMAA.

## Results

### Market basket study

**Figure 1:**
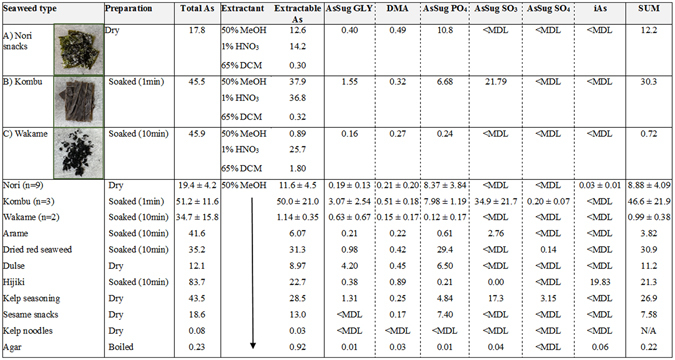
Arsenic speciation for seaweeds used in feeding study (**A**–**C** at the top of the table) and in 23 seaweeds and seaweed products marketed for consumption. Concentrations of total arsenic, extractable arsenic (50% methanol (MeOH); for seaweeds (**A**–**C**): 1% HNO_3_ heated to 90 °C, 65% dichloromethane (DCM) also shown) and arsenic species are all reported in µg/g.

A range of arsenic concentrations and species were detected in the 22 seaweeds and seaweed products tested from local markets; seaweeds were prepared (raw/soaked) according to consumption guidelines prior to analysis (Fig. 1). Brown algae products (hijiki, kombu, wakame, arame; 45.0 ± 22.2 µg/g) had higher arsenic concentrations (*p* = 0.004) than red algae (nori, red seaweed; 19.2 ± 8.4 µg/g), with hijiki containing the highest concentrations (83.7 µg/g). The seaweed extract products, agar agar and kelp noodles, had relatively low arsenic concentrations (<1 µg/g). Extraction efficiency (arsenic in 50% methanol extract *vs*. arsenic in digested sample) varied, and was particularly low for wakame (2–6%). Using a more aggressive extraction procedure (weak acid with microwave heating) to further characterize the seaweeds (nori (A), kombu (B), and wakame (C)) used in the feeding study gave higher, but still incomplete, recovery for seaweed C (56%). Low levels of lipophilic arsenic were extracted in methanol: dichloromethane (DCM) from seaweeds A and B, with a slightly higher concentration in seaweed C (1.8 µg/g).

Inorganic arsenic concentrations were negligible in all seaweed samples except hijiki, for which 87% of the extractable arsenic was present as iAs. Arsenosugars were the major species in all other seaweeds, along with trace amounts of DMA (<1 µg/g). In nori, arsenosugar-phosphate (PO_4_) was the predominant form of arsenosugar, and arsenosugar-sulfonate (SO_3_) was most prevalent in kombu. The low extraction efficiency in wakame made arsenic speciation of this seaweed difficult to assess. Speciation of the weak acid digestion showed 96% of arsenic to be present as arsenosugar, with arsenosugar-glycerol (GLY) as the major form of arsenic in wakame. However, at higher temperatures and acidity, some interconversion between arsenosugar species occurs, as well as formation of the breakdown product arsenosugar-OH, which is not chromatographically separated from arsenosugar-GLY^25,26^. This leaves uncertainty in speciating arsenosugars in weak acid extracts.

### Feeding study

**Figure 2:**
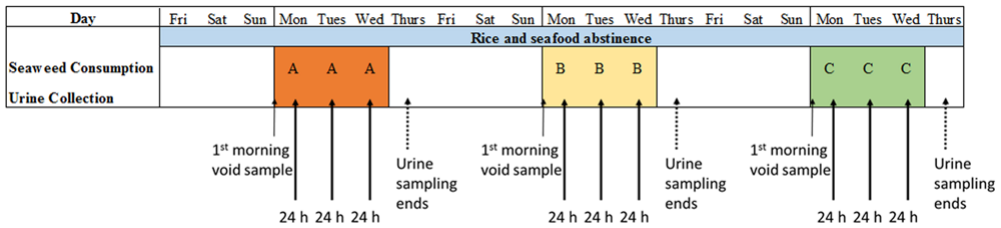
Feeding experiment design.

Of the 11 participants in the seaweed consumption experiment, 7 were women and 4 were men; ages ranged from 24 to 61, and body mass indexes from 18.4 to 28.5. Three days prior to the experiment, and throughout the feeding blocks, participants refrained from eating seafood and rice. Three feeding blocks were completed by each participant, in which participants consumed 10 g portions of seaweeds (A, B, or C) each day and collected 24 h urine samples (see Fig. 2). Seaweed A was consumed uncooked, and seaweeds B and C were soaked in 125 mL water for 1 (B) or 10 (C) minutes, then the water discarded (Fig. 1). For each feeding block, participants collected a morning void urine sample prior to seaweed consumption to establish a baseline urinary arsenic concentration (D0). The three 24 h collections began at the time of the seaweed was consumed (D1-D3).

**Figure 3:**
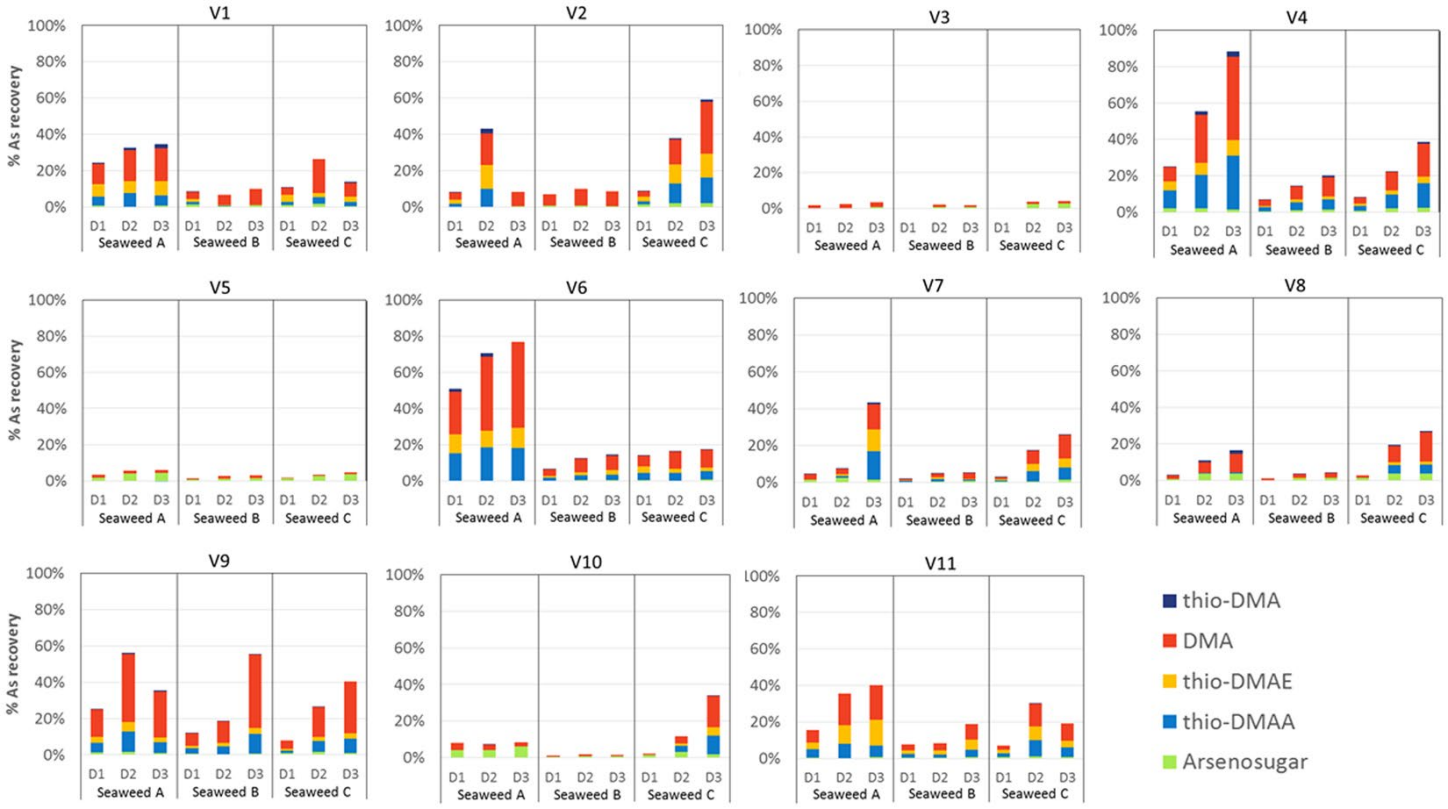
Concentrations of arsenic species (µg/L) in urine samples, normalized to specific gravity. Samples labelled D0 (day 0) are spot samples on the day prior to seaweed consumption, and D1 (day 1), D2 (day 2) and D3 (day 3) are 24 h urine collection samples for the days following consumption of each seaweed portion. Seaweeds A, B and C were nori, kombu and wakame, with arsenic concentrations of 17.1, 45 and 46 µg/g respectively. Major arsenic species (DMA, thio-DMAE, thio-DMAA), intact arsenosugars (sum of As sugar-GLY, -PO_4_ and -SO_3_), and thio-DMA are shown; see legend for color codes. Figure 2 Inset Mean before ingestion-after ingestion differences in urinary concentrations of arsenic species and total arsenic across individuals for each seaweed type. Arsenic species are color-coded according to the legend. Before-after differences were adjusted for age, gender and BMI. Symbols represent statistical significance (^+^represents p < 0.05; *represents p < 0.001).

Concentrations of total urinary arsenic were generally low (undetectable, <0.4 µg/L, to 9.9 µg/L) at baseline (D0). Mean urinary arsenic concentrations across all individuals increased significantly following consumption of all seaweed types (*p* < 0.05 in generalized estimating equations (GEE) models adjusted for age, gender and body mass index (BMI)) (Fig. 3 inset). Mean increases were 20.5 ± 14.4, 15.2 ± 11.0 and 37.8 ± 14.2 µg/L arsenic for seaweeds A, B and C respectively.

Before treatment (D0), arsenosugars and their metabolites thio-DMAA and thio-DMAE were undetectable for all individuals (Fig. 3). Low levels of DMA and trace amounts of thio-DMA were detectable in some baseline urine samples. Following seaweed consumption, increases were observed for DMA (mean 7.9, 7.0, and 14.1 µg/L for seaweeds A–C, respectively), thio-DMAA (mean 6.1, 3.5, and 9.9 µg/L for A–C) and thio-DMAE (mean 4.5, 2.2, and 7.6 µg/L for A–C). Small increases in intact arsenosugars, reported as the sum of arsenosugar-GLY, PO_4_, and SO_3_, were observed (mean 1.7, 1.6, 3.2 µg/L for A–C); arsenosugar- SO_3_ was only detected following consumption of seaweed B, whereas arsenosugars GLY and PO_4_ were detected at low levels following consumption of all three seaweeds. Thio-DMA, the known cytotoxic metabolite, increased in some individuals following seaweed consumptions, however, it was detected at relatively low concentrations (maximum of 6.6 µg/L) and the difference did not reach statistical significance (Fig. 3 inset).

Individual excretion varied with two individuals (V3, V5) experiencing almost no change in urinary arsenic levels before and after the treatment, while four excreted over 100 µg/L arsenic in 24 h samples (V4, V7, V9) (Fig. 3). Variability in urinary arsenic concentrations between individuals could not be explained by age or BMI, and gender was only found to be a predictor of thio-DMAE excretion, with relatively higher concentrations in men than women (β = 5.20, 95% CI: 2.25, 8.15). Between person variability was 23%, 35%, 12% of the total variance for seaweeds A, B and C respectively, after adjustment for the treatment status (i.e., whether the sample was taken before or after seaweed consumption), age, gender and BMI. Arsenic concentrations often increasing from Day 1 to Day 3 although not consistently so. Likewise, individual differences varied by type of seaweed (nori, kombu, wakame (A–C)) consumed: some individuals (V2, V4, V8, V10) excreted the highest urinary arsenic concentrations following wakame consumption, but another did so (V9) after eating kombu, despite similarity in arsenic concentrations in these seaweeds (45.5, 45.9 µg/g arsenic, respectively). The concentration of arsenic in nori (17.8 µg/g) was lower than for other seaweeds, but higher levels of arsenic were excreted by several individuals following nori consumption (V4, V7, V11) than from kombu. This was further investigated by estimating recovery of arsenic following consumption of different seaweed types.

### Recovery of arsenic from seaweed

The total amount of arsenic from seaweed in each 24 h urine sample was first determined to correct for the large variability in sample volume between individuals, and to a lesser extent, between days. The concentration of each arsenic species in the 24 h sample was multiplied by the sample volume to give the amount of arsenic in each sample (µg) (Supplemental materials, Fig. 1S). The recovery of arsenic from consumption of different seaweeds was then estimated by dividing the amount of each arsenic species in the 24 h urine sample (µg) by the amount of arsenic in the daily seaweed portion consumed (178, 455, and 459 µg for seaweeds A, B and C, respectively). Because the retention times of arsenosugars can be long (>24 h)^4^, arsenic ingested on D1 likely continues to be excreted on D2 and D3, along with those days’ intakes. Mean recoveries of arsenic species for each individual and feeding block were calculated across days (averages of D1 to D3), to partially account for this carryover. These values are estimates because not all recovery can be accounted. Estimated mean recoveries among all individuals for each seaweed (±standard deviation) are presented in Table 1.

**Table 1:** Mean estimated recovery (±standard deviation (SD)_of arsenic species in urine (relative to total arsenic consumed) for each feeding block.

	As Sugar	Thio DMAA	Thio DMAE	DMA	Thio DMA	Total As
**Seaweed A**	1.6 ± 1.6%	6.0 ± 6.7%	4.2 ± 3.8%	13.6 ± 11.7%	0.6 ± 0.6%	27.7 ± 20.6%
**Seaweed B**	0.7 ± 0.4%	1.5 ± 2.1%	0.9 ± 1.2%	5.3 ± 5.4%	0.1 ± 0.2%	10.5 ± 9.1%
**Seaweed C**	1.7 ± 0.8%	4.2 ± 2.9%	2.8 ± 2.4%	8.6 ± 4.8%	0.2 ± 0.3%	19.6 ± 9.9%
**P value**	<0.001	<0.001	<0.001	<0.001	<0.001	<0.001

Differences in mean recovery between seaweeds were statistically significant (*p* < 0.001) for total arsenic and all urinary arsenic species (Table 1). While urinary arsenic concentrations from equivalent portions of seaweed were highest following consumption of wakame (C), recovery from nori (A,) that has the least arsenic, was highest. Kombu (B) and wakame (C) had similar arsenic concentrations, yet arsenic recovered in urine was significantly higher from the wakame. Differences in recovery of major metabolites, DMA, thio-DMAA and thio-DMAE (from total arsenic consumed), followed the same pattern, where recovery was highest in seaweed A > C > B. Despite the higher bioavailability of arsenic from nori (A), the higher concentrations of arsenic present in wakame (C) resulted in higher urinary arsenic concentrations from an equivalent portion of seaweed consumed, whereas the low bioavailability of arsenic in kombu (B) resulted in similar mean urinary arsenic concentrations to nori (A) (Fig. 3 inset), which had a 50% lower arsenic concentration.

### Correlations between metabolites

Correlations between urinary arsenic species were determined to ascertain whether concentrations of seaweed specific thio-DMAE and thio-DMAA were associated with concentrations of DMA and other common species across samples. If so, it would suggest that these metabolites would reflect concentrations of DMA and total arsenic coming from seaweed consumption. Urinary total arsenic, and the major metabolites, DMA, thio-DMAE and thio-DMAA were all strongly correlated, whereas arsenosugar concentrations did not correlate with other arsenic species (Table 2). Correlation coefficients were similar for total arsenic *vs*. DMA for all three seaweeds (A, B, C: *r*^*2*^ = 97, 97, 96%), whereas there were some differences in the correlations of thio-DMAA and thio-DMAE with arsenic and DMA: correlations between thio-DMAE and thio-DMAA with total arsenic (TAs) and DMA concentrations were weaker for kombu (B) (thio-DMAE *vs*. TAs: 62%; thio-DMAE *vs*. DMA: 64%; thio-DMAA *vs*. TAs: 65%; thio-DMAA *vs*. DMA: 69%) than for nori (A) and wakame (C) (thio-DMAE *vs*. TAs: 86%(A), 84%(C); thio-DMAE *vs*. DMA: 79% (A), 81% (C); thio-DMAA *vs*. TAs: 93%(A), 93%(C); thio-DMAA *vs*. TAs: 89%(A), 91%(C)).

**Table 2:** Correlations between arsenic species in urine.

	AsSugar	Thio DMAA	Thio DMAE	DMA	Thio DMA	TAs
**AsSugar**	100%					
**Thio DMAA**	12%	100%				
**Thio DMAE**	1%	90%	100%			
**DMA**	7%	83%	75%	100%		
**Thio DMA**	28%	77%	70%	61%	100%	
**TAs**	9%	84%	78%	97%	60%	100%

## Discussion

We found high levels of arsenic in commercially available seaweeds, primarily in the form of arsenosugars. Consumption of seaweeds led to urinary arsenic reaching higher levels than attributed to other staple sources of arsenic. Increases in the unique arsenosugar metabolites thio-DMAA and thio-DMAE, were observed along with DMA and intact arsenosugars in urine, with strong correlations between excreted species. Urinary arsenic concentrations varied between individuals, between seaweed types, and in individual response to treatment (seaweed type).

In agreement with previous studies, products made from red algae had lower arsenic levels than those made from brown algae^17,18^. Concentrations in products based on whole seaweeds ranging from 12.1 to 83.7 µg/g, whereas seaweed extract products had much lower concentrations (<1 µg/g). As expected, hijiki was the only seaweed in which iAs was detected, and the very high concentrations of iAs in this species have been deemed a significant exposure risk^27,28^. Low extraction recoveries were observed for some seaweeds, particularly wakame, which has been reported previously^16^. For most seaweeds, arsenic was almost completely in the form of arsenosugars, along with trace levels of DMA. Seaweeds are a significant source of arsenosugar intake, but measurement of the food source alone does not give a complete picture of exposure.

In our experimental feeding study of three seaweed products, urinary arsenic excretion patterns were similar to those previously found with purified arsenosugar intake^3,4^, but not to those reported in the Chinese study, which as mentioned had limitations^24^. The major product of arsenosugar metabolism, DMA, is also formed from iAs and arsenolipid metabolism^29,30^, and can be a component of arsenic in food, i.e., rice^31^. The thio-analog of DMA also has been observed in urine samples from a population exposed to high levels of iAs through drinking water^32^. Thus it was not surprising that low levels of DMA, and occasionally thio-DMA, were detected in the baseline samples of some individuals in our study due to intake of DMA from the diet, or from metabolism of iAs from drinking water or food. While other sources of DMA clearly exist, urinary concentrations of DMA increased more than other arsenic compounds following seaweed consumption. By contrast, thio-DMA levels increased slightly in a few individuals following seaweed consumption, but this was not statistically significant across volunteers. Thus, while DMA is not specific to arsenosugar metabolism, seaweed is a potential source of high levels of DMA detected in urine. Unlike DMA, the metabolites thio-DMAA and thio-DMAE, have been only associated with the breakdown of arsenosugars^3^, and are not known to be present in appreciable quantities in other foods. Arsenosugars also can be found in low levels in shellfish that feed on algae. In our study, subjects abstained from seafood prior to the experiment, and baseline concentrations of arsenosugars, thio-DMAA and thio-DMAE were consistently below detection limit. All three arsenic species increased in urine after seaweed consumption, with much higher concentrations of the metabolites than of intact arsenosugars. This suggests compounds thio-DMAA and thio-DMAE may provide unique markers of arsenosugar metabolism.

Mean urinary arsenic concentrations we observed following seaweed consumption were higher than levels associated with drinking water at the EPA guideline of 10 µg/L, and similar to concentrations associated with a high rice diet (6 individual fed 300 g rice *d.w*. per day had mean urinary arsenic of 50 µg/L in first pass/spot samples after 4 days)^33^. The concentrations of urinary arsenic from seaweed consumption therefore likely overwhelm arsenic from other sources. Despite individual variability in urinary arsenic concentrations, thio-DMAE and thio-DMAA were strongly correlated with total arsenic and DMA. Correlations between thio-DMAE and thio-DMAA and the other metabolites were slightly lower for kombu than the other seaweed species, and may be related to different retention rates observed for these compounds^3,4^. Still, similar behavior between arsenosugar metabolites across samples suggests thio-DMAE and thio-DMAA can be used to distinguish arsenic from seaweed consumption relative to other sources.

Repeated feeding studies provide a realistic model of dietary intake; in this study individual arsenic excretion was variable, and often increased, between days in each feeding block. Retention times of arsenic following seaweed^23,34–36^ or arsenosugar consumption^3,4,37^ are longer than those observed for inorganic arsenic^38,39^, where peak arsenic excretion levels have been found to be between 9 to 60 h following intake, compared to 4–14 h for inorganic arsenic. Thus, in our study, arsenic from seaweed consumed on Day 1 likely was still being excreted on Days 2 and 3, resulting in higher urinary arsenic levels after repeated feedings. Repeated consumption of seafood meals containing seaweed also has been shown to result in substantially higher urinary arsenic concentrations relative to a single dose^40^.

Variability in arsenic excretion was observed both between individuals and between seaweed types. Inter-individual variability in urinary arsenic levels following seaweed consumption has been reported previously^34^, and individual recoveries ranged from 4–95% following ingestion of a pure arsenosugar^4^. The reason for individual variability is unclear. We did not find individual factors tested (e.g., gender, BMI, and age) explained the variability in arsenic excretion across feeding blocks. The absorption and metabolism pathways of organic arsenic are not fully known, but current understanding is that if arsenic absorbed in the gut the majority of it is excreted in via the kidneys in urine, whereas arsenic that is not absorbed is excreted in feces^41^. One hypothesis for explaining variability in urinary arsenic concentrations is that there may be individual differences in absorption or breakdown in the gut^4^. Factors such as the gut microbiome composition, as well as genetic differences affecting enzyme production, or lifestyle factors could potentially influence arsenic absorption. These were not measured in our study and will need to be investigated in the future.

We also found differences in urinary excretion of arsenic by type of seaweed consumed. Higher arsenic bioaccessibility, using an *in vitro* method, has been reported for nori and kombu relative to wakame^28^. The low extraction efficiency from wakame in our study suggests arsenic from this seaweed has lower solubility than other types. Yet arsenic recovery from wakame ingestion was lower than for nori but not kombu, indicating these extraction methods are not strong predictors of arsenic concentrations in urine. The metabolites present in urine following consumption of wakame were similar to the other seaweeds suggest arsenic in the un-extractable fraction is in the form of arsenosugars. Arsenosugar species have been shown to have different gut permeability and cellular bioavailability in *in vitro* incubation experiments^7^. The different predominant arsenosugar species in seaweeds A–C could contribute to differences in bioavailability, and to individual variability in enzyme activity favoring metabolism of different arsenosugars. The presence of arsenolipids in some seaweeds^42–44^, may also contribute urinary arsenic^29^. A small amount of lipid-soluble arsenic was detected in the wakame samples, but concentrations of this species alone are too low to explain the urinary arsenic levels observed in volunteers who only excreted arsenic from consumption of this seaweed. Overall, differences in arsenic bioavailability between seaweed types are evident, but variability in individual response also occurs, implying individual exposure is best assessed by urinary markers.

Only limited toxicity testing exists on arsenosugars and their metabolites^45^. Recent *in vitro* studies found arsenosugars, as well as the metabolites thio-DMAE and thio-DMAA have low cytotoxicity in bladder cells^7^, whereas thio-DMA has similar or higher cytotoxic than inorganic arsenic^5–7^. Thio-DMA has been shown to be relatively stable over time (1% or less degradation per week at r.t.; 10% at 60 °C)^46^, so once formed, appreciable degradation of the thio-compound *in vivo* is unlikely. In our study and investigations of synthetic arsenosugars^3,4^, only low concentrations of thio-DMA were excreted, suggesting this is formed at minimal levels during arsenosugar metabolism. However, further toxicological evaluations are needed to properly assess risk associated with seaweed consumption.

In summary, seaweeds available to consumers have variable amounts and bioavailability of arsenic, and individual response to seaweed ingestion also varied. Ingestion of commonly consumed seaweeds increased levels of arsenosugars and metabolites, including the unique products thio-DMAA and thio-DMAE, whereas concentrations of the known toxic metabolite, thio-DMA were detectable only at low concentrations. Assessment of thio-DMAA and thio-DMAE as urinary biomarkers may provide a means of characterizing intake of arsenosugars from other arsenic species and evaluating the health impacts from exposure to these compounds in the future.

## Methods

### Market basket study

Twenty-three seaweeds and seaweed products marketed for consumption and chosen to reflect a range of available products, were purchased from a supermarket in Lebanon, New Hampshire, USA (Fig. 1). Dried samples were homogenized with a stainless steel food processor prior to digestion/extraction. Seaweeds were then prepared according to instructions for consumption prior to analysis; samples were either digested and extracted in their dried form, or soaked in cold water (10 g seaweed in 125 mL water) for 1 or 10 minutes then the water discarded, or boiled for 5 minutes (4 g in 1 L of water) (see Fig. 1). Wet and dry weights were recorded, and all concentrations were converted to dry weight for comparison.

### Feeding study

Eleven volunteers were given written instructions and all materials needed for seaweed consumption (pre-weighed 10 g portions of seaweed) and urine collection (vials). Subjects understood the project and experimental details, and the experimental protocol was approved by the Dartmouth College Institutional Review Board (IRB) for human subject protection. Written, informed consent was received from each of the participants prior to the study, and all protocols were performed in agreement with guidelines and regulations specified by the IRB.

The experimental feeding study design is outlined in Fig. 2. Baseline spot samples (D0) were collected in acid-washed 250 mL vials prior to each feeding block. Urine samples (D1 to D3) were then collected in acid-washed 500 mL bottles, then composited into one (or more) 3 L containers for each 24 h period (e.g., immediately following seaweed consumption on Day 1 until the same time on Day 2). Samples were kept cool then delivered to the laboratory each day. Specific gravity and pH were measured, and subsamples were taken for arsenic analysis in 15 mL vials, and frozen to −20 °C.

### Total As determination in seaweeds

Analytical protocols for determining arsenic in seaweed were reported previously^17^. Seaweed samples (0.25 g) were weighed into 10 mL Teflon vessels and 5 mL concentrated HNO_3_ (sub-boil distilled Fisher Trace Metal Grade) was added to each vessel. Samples were heated to 190 °C for 10 min by high pressure microwave digestion (MARS XPRESS, CEM, Mathews, NC), then transferred to pre-weighed 60 mL vials (Sarstedt, Germany), and diluted to 50 mL with ultrapure water (18 MΩ cm, EMD Millipore, Darmstadt, Germany), and weighed. The samples were diluted (by mass) another 20-fold with 1% HNO_3_ into 7 mL vials for analysis. Total arsenic was determined by collision cell ICP-MS (7700x, Agilent, Santa Clara, CA) with a collision gas flowrate of 5 ml/min He. Recoveries of arsenic in digested standard reference materials (SRM) analyzed along with sample batches were 102 ± 13% (*n* = 2) for DOLT-4 Dogfish Liver (9.66 mg/kg; NRC, Ottawa, Canada), 103% (*n* = 1) for NIST 1566b Oyster tissue (7.65 mg/kg; Gaithersburg, MD), and 90 ± 7% (*n* = 2) for an in-house kelp standard from a Brooks Rand Laboratory inter-laboratory comparison (63.7 mg/kg)^47^.

### Extraction and As speciation analysis in seaweeds

Briefly, 10 mL of 1:1 v/v methanol:water was added to 0.5 g seaweed; samples were sonicated for 1 h, left overnight, then centrifuged. The supernatant was removed and evaporated, then re-diluted in 10 mL water. The sample extract was filtered through a 0.2 µm pore-size syringe filter (Whatman, Maidstone, UK), and transferred to a 1 mL PTFE septa vial (Agilent). Another aliquot of the final solution was diluted 20-fold with 1% HNO_3_ and analyzed, as above, for total extractable arsenic.

To further characterize the three samples used in the feeding study, samples were also extracted in 1% HNO_3_, heated to 90 °C for 6 min^48^, and analyzed for total arsenic and arsenic species, to attain a higher recovery of arsenic. An extraction in 2:1 dichloromethane: methanol was performed to determine the lipid soluble fraction of arsenic in these seaweeds. Recovery of lipid-soluble arsenic from certified reference material (CRM 7405-a (Hijiki), was higher in this study (9 µg/g) *vs*. that reported previously (6.2 µg/g)^43^.

Speciation analysis was performed on an Agilent LC1120 liquid chromatograph coupled to collision cell ICP-MS, using an anion exchange column (Hamilton PRP-X100 10 µm 4.6 × 250 mm; Reno, NV) with a mobile phase of 20 mM (NH_4_)_2_CO_3_ at pH 9, a column temperature of 40 °C and a flow rate of 1.5 ml min^−1^. The ICP-MS was operated in collision mode with He as the collision gas at a flow rate 2 ml min^−1^. Inorganic As3 and As5 were obtained from inorganic ventures (Christiansburg, VA), monomethyl sodium arsonate, MA, dimethylsodium arsenate, DMA, (Chem Service, West Chester, PA) arsenobetaine, AB, (Sigma Aldrich, St Louis, MO), and arsenosugars were isolated and donated by Jack Creed at U.S.EPA. An additional separation method was used to verify identification of cationic species which were not strongly retained on the anion exchange column; species were separated on a Supercosil SCX (5 µm 4.6 × 250 mm; Sigma Aldrich, St. Louis, MO) column with a mobile phase of 20 mM pyridine at pH 2.5, and a column temperature of 40 °C. Recovery of AB (3.95 mg/kg) in DORM-4 (NRC) was 94 ± 8% (n = 3). Speciation of arsenic in MURST-ISS-A2 found in this study (in mg/kg, *n* = 3) was comparable to literature values (Grotti *et al*., 2010), given in italics: AsSugar- PO_4_ = 0.31 ± 0.02 (*0.3*), AB = 1.84 ± 0.06 (*1.9*); DMA = 0.26 ± 0.01 (*0.22*).

### Urine Analyses

Samples were thawed immediately prior to analyses, then vortexed. An aliquot (0.5 mL) of each samples was diluted 10-fold with 1% HNO_3_ for total arsenic analysis. A second aliquot (0.2 mL) was taken, after sediment was allowed to settle, and diluted to 1 mL with ultrapure water (18 MΩ cm, EMD Millipore, Darmstadt, Germany) for arsenic speciation analysis. Samples were analyzed for total arsenic by triple quad ICP-MS (8800 ICP-QQQ; Agilent, Santa Clara, CA) operated with oxygen as a reaction gas (0.3 mL/min) and measurement of arsenic as AsO^+^ at m/z 91. Quality control was assessed using calibration check standards, which were analyzed every 10 samples throughout each run (92–101% recovery), and duplicate and standard spike samples were monitored every 20 samples.

For speciation analyses, a chromatography system (Agilent 1260, Santa Clara, CA) was interfaced directly to the 8800 ICP-QQQ (Agilent, Santa Clara, CA). To determine all of the As species and metabolites present in urine, three different chromatographic methods were employed, similar to those discussed in previous studies^3,49,50^. Thiolated As species were analyzed by reverse phase chromatography using an Atlantis dC18 column (Waters, Milford, MA) with 1 mL/min 20 mM NH_4_ formate at 30 °C as the mobile phase^50^. Samples were then treated with 10% H_2_O_2_ and analyzed by anion exchange chromatography (Hamilton PRP X100) for the determination of iAs, DMA, DMAA and arsenosugars – PO_4_, SO_3_, and SO_4_; and cation exchange was used for determination of arsenosugar-GLY, DMAE and AB, using the conditions described above. Incomplete resolution of thio-DMAA and thio-arsenosugar-SO_3_, as well as thio-DMAE and thio-arsenosugar-PO_4_, can occur by the C18 separation, but concentrations of arsenosugars are small relative to their metabolites (thio-DMAA and thio-DMAE)^50^, which was confirmed by total arsenosugar analysis (in the oxidized sample).

In addition to standards listed above, DMAE and DMAA were synthesized and characterized by K.A. Francesconi (U. Graz); thio-analogs of DMAE, DMAA and DMA, as well as arsenosugars, were produced by reaction with H_2_S, as described previously^50^. Quality control was determined by repeated analysis of NIST 2669 Arsenic Species in Urine (Level 1 and Level 2 were alternated every 10 samples), and by duplicate and spike recoveries throughout each run. By the C18 method, recoveries in the NIST standards were 92–123% for DMA (*n* = 17) and 91–117% for AB (*n* = 9, Level 1 only). Recoveries of AB, DMA, MA, As5 in the NIST standards were 85–120% by anion exchange chromatography (*n* = 17), recoveries; and recoveries of DMA, AB and arsenocholine (AC) were 89–110% by cation exchange chromatography (*n* = 12). The limits of detection (LOD) for arsenic species by this method were 0.02 µg/L in diluted (5X) urine, for all species except As5 for which the detection limit was 0.05 µg/L.

### Statistical analysis methods

Descriptive statistics such as mean, standard deviation, median and interquartile range were calculated for all continuous variables and frequencies for categorical or binary variables. Analytes that were less than detection limit were assigned a value of LOD/(√2), with the exception of the arsenosugars and their unique metabolites (thio-DMAE, thio-DMAA), which were assigned a value of zero, because their presence is not expected in baseline samples (prior to seaweed consumption). Two baseline spot samples (D0) were missing (V10 and V11 for seaweed B); an average of the D0 values from feeding blocks for seaweeds A and C were assigned for these samples.

The generalized estimating equations (GEE) approach^51^, a semiparametric regression method, was used to estimate and test the difference between baseline (before seaweed consumption) and the average arsenic species levels for the 3 days of consumption (i.e., before versus after treatment) for each species and each seaweed type. An autoregressive (order 1) working correlation structure was used in the GEE approach to model the within-subject correlations. Age, gender and BMI were adjusted in these models. Variance component analysis was performed to derive the proportion of within-subject and between-subject variance after adjusting for treatment effects and covariates.

The GEE equation of the regression model for the mean is given in Equation 1, where $${{\rm{y}}}_{{\rm{ij}}},\,{\rm{i}}=1,\ldots ,11,$$$$\,{\rm{j}}=1,\ldots ,4$$ and denotes the arsenic concentration level for the ith person at the jth measurement; j = 1 indicates the baseline measurement and the j = 2, 3, 4 indicates the three follow-up measurments.

1 $${\rm{E}}({{\rm{y}}}_{{\rm{ij}}})={{\rm{\beta }}}_{0}+{{\rm{\beta }}}_{1}{\ast \mathrm{trt}}_{{\rm{ij}}}+{{\rm{\beta }}}_{2}{\ast \mathrm{age}}_{{\rm{i}}}+{{\rm{\beta }}}_{3}{\ast \mathrm{sex}}_{{\rm{i}}}+{{\rm{\beta }}}_{4}{\ast \mathrm{BMI}}_{{\rm{i}}},$$

E(y_ij_) is the mean value of y_ij_, β_0_ is the intercept, β_1_ is the treatment effect (or before-after treatment difference), β_2_, β_3_ and β_4_ are the effects of age, sex and BMI respectively. The treatment variable trt_ij_ takes value of 0 at baseline (ie. i = 1) and 1 at follow up time points (ie. i = 2, 3, 4). The GEE approach assumes the correlations for the four repeated measurements have an autoregressive (AR1) structure meaning that ${\rm{corr}}({{\rm{y}}}_{{\rm{ik}}},{{\rm{y}}}_{{\rm{il}}})={{\rm{\rho }}}^{|{\rm{l}}-{\rm{k}}|},\,1\le {\rm{k}},{\rm{l}}\le 4$. Under this correlation structure, any two adjacent measurements have the same correlation, and the further two measurements apart, the weaker the correlation.

Recovery of arsenic was calculated from urinary concentrations of seaweed-related arsenic species measured in 24 h samples multiplied by the volume of urine and divided by the concentration of arsenic in the seaweed consumed. Mean recovery was reported across days for each seaweed type. Correlation matrices were calculated for arsenic metabolites, arsenosugars and total arsenic for all days and seaweed types, and the average correlation values across days and seaweed types were presented.

## Electronic supplementary material

Supplementary materials
