# Author Correction: Distinct arsenic metabolites following seaweed consumption in humans

**DOI:** 10.1038/s41598-018-22625-x

**Published:** 2018-03-02

**Authors:** Vivien F. Taylor, Zhigang Li, Vicki Sayarath, Thomas J. Palys, Kevin R. Morse, Rachel A. Scholz-Bright, Margaret R. Karagas

**Affiliations:** 1 Department of Earth Science, 6105 Sherman Fairchild Hall, Dartmouth College, Hanover, NH 03755 USA; 2 Department of Epidemiology, Geisel School of Medicine, 1 Medical Center Drive 7927 Rubin Building, Lebanon, NH 03756 USA

Correction to: *Scientific Reports* 10.1038/s41598-017-03883-7, published online 20 June 2017

This Article contains errors in Figure 3. In Figure 3A the y-axis ‘As (µg/L)’ is incorrectly given as ‘% As recovery’.

In addition, Figure 3B was omitted.

Finally, the Figure legend,

“Figure 2 Inset Mean before ingestion”

should read

“B) Mean before ingestion”

The correct Figure 3 appears below as Figure 1.

**Figure 1:**
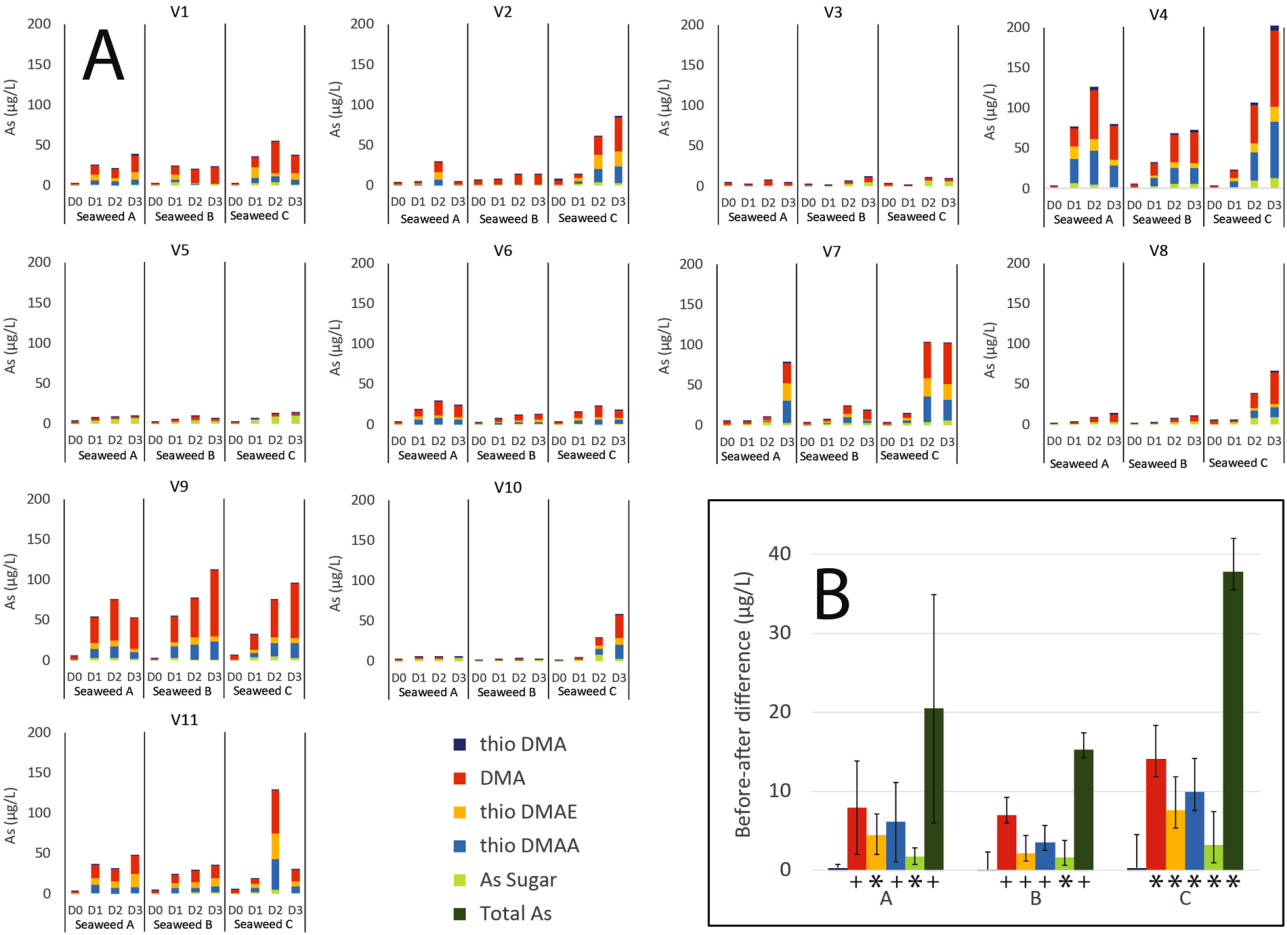
(**A**) Concentrations of arsenic species (µg/L) in urine samples, normalized to specific gravity. Samples labelled D0 (day 0) are spot samples on the day prior to seaweed consumption, and D1 (day 1), D2 (day 2) and D3 (day 3) are 24 h urine collection samples for the days following consumption of each seaweed portion. Seaweeds A, B and C were nori, kombu and wakame, with arsenic concentrations of 17.1, 45 and 46 µg/g respectively. Major arsenic species (DMA, thio-DMAE, thio-DMAA), intact arsenosugars (sum of As sugar-GLY, -PO_4_ and -SO_3_), and thio-DMA are shown; see legend for color codes. (**B**) Mean before ingestion-after ingestion differences in urinary concentrations of arsenic species and total arsenic across individuals for each seaweed type. Arsenic species are color-coded according to the legend. Before-after differences were adjusted for age, gender and BMI. Symbols represent statistical significance (^+^represents p < 0.05; *represents p < 0.001).

