# Supplementary material for: Distinct arsenic metabolites following seaweed consumption in humans
Source: Sci Rep. 2017 Jun 20;7:3920. doi: 10.1038/s41598-017-03883-7 (PMC5478658; doi:10.1038/s41598-017-03883-7)

Supplemental material:

Distinct arsenic metabolites following seaweed consumption in humans

Vivien F. Taylor [a], Zhigang Li [b], Vicki Sayarath [b], Thomas J. Palys [b], Kevin R. Morse [b], Rachel A. Scholz-Bright [a], Margaret R. Karagas [b]

a) Department of Earth Science, 6105 Sherman Fairchild Hall, Dartmouth College, Hanover, NH, USA 03755
b) Department of Epidemiology, Geisel School of Medicine, 1 Medical Center Drive
7927 Rubin Building, Hanover, NH, USA, 03756

Corresponding author: Vivien F. Taylor, Department of Earth Science, 6105 Sherman Fairchild Hall, Dartmouth College, Hanover, NH, USA 03755; 603-646-3318; vivien.f.taylor@dartmouth.edu

Table 1S: Summary of literature studies on seaweed and arsenosugar consumption.

**Reference**	**Seaweed/Compound**	**Subjects**	**Intervention**	**Urine collection**	**Dietary abstinence**	**Recovery**	**Urinary arsenic species observed[a]**
Le, 1994	nori, kelp powder	9	1feeding (9.5 g)	spot samples for 4 d	seafood for 3 d prior + during expt	N/A	Uncharacterized
Ma and Le, 1998	nori	4	2 feedings (10 g)	spot samples for 3 d	seafood for 3 d prior + during expt	N/A	DMA
Francesconi, 2002	synthetic arsenosugar	1	1 feeding (1220 µg As)	24h samples for 4 d	seafood, mushrooms for 4 d prior + during expt	80%	DMA and specific
Wei, 2003	nori	6	1 feeding (15 g)	spot samples for 4 d	seafood for 3 d prior + during expt	N/A	DMA
Van Hulle, 2004	kombu	5	1 feeding (20-25 g)	24h samples for 5 d	seafood for 3 d prior + during expt	N/A	DMA and specific
Matsuura, 2005	hijiki	1	1 feeding (15 g)	spot samples for 3 d	high As food during expt	N/A	DMA
Raml et al., 2005	synthetic arsenosugar	1	1 feeding (945 µg As)	24h samples for 4 d	seafood, mushrooms for 4 d prior + during expt	81%	Extensive
Raml et al., 2009	synthetic arsenosugar	6	1 feeding (850-1100 µg As)	24h samples for 4 d	seafood for 3 d prior + during expt	4-95%	Extensive
Choi et al., 2012	kelp and laver (& flatfish, conch)	16	6 feedings (seafood meal)	spot samples for 14 d	seafood for 3 d prior + during expt	N/A	DMA
Hata, 2013	wakame	5	1 feeding (300 g)	24h samples for 5 d	seafood for 5 d prior + during expt	9-41%	DMA
Wang et al., 2015	Chinese seaweed (unspecified)	10	1 feeding (20g)	spot samples for 3 d	high As foods for 1 week prior + during expt	N/A	DMA and specific

a) Urinary species detected: DMA; DMA and specific (DMA and one or more metabolites specific to arsenosugar metabolism (eg. oxo/thio-DMAE, oxo/thio-DMAA); Extensive (characterization included oxo/thio arsenosugar, oxo/thio-DMA, oxo/thio-DMAE, oxo/thio-DMAA, TMAO).

Fig. 1S Total amounts of arsenic species (µg) in 24 h urine samples, calculated from concentrations multiplied by sample volume.

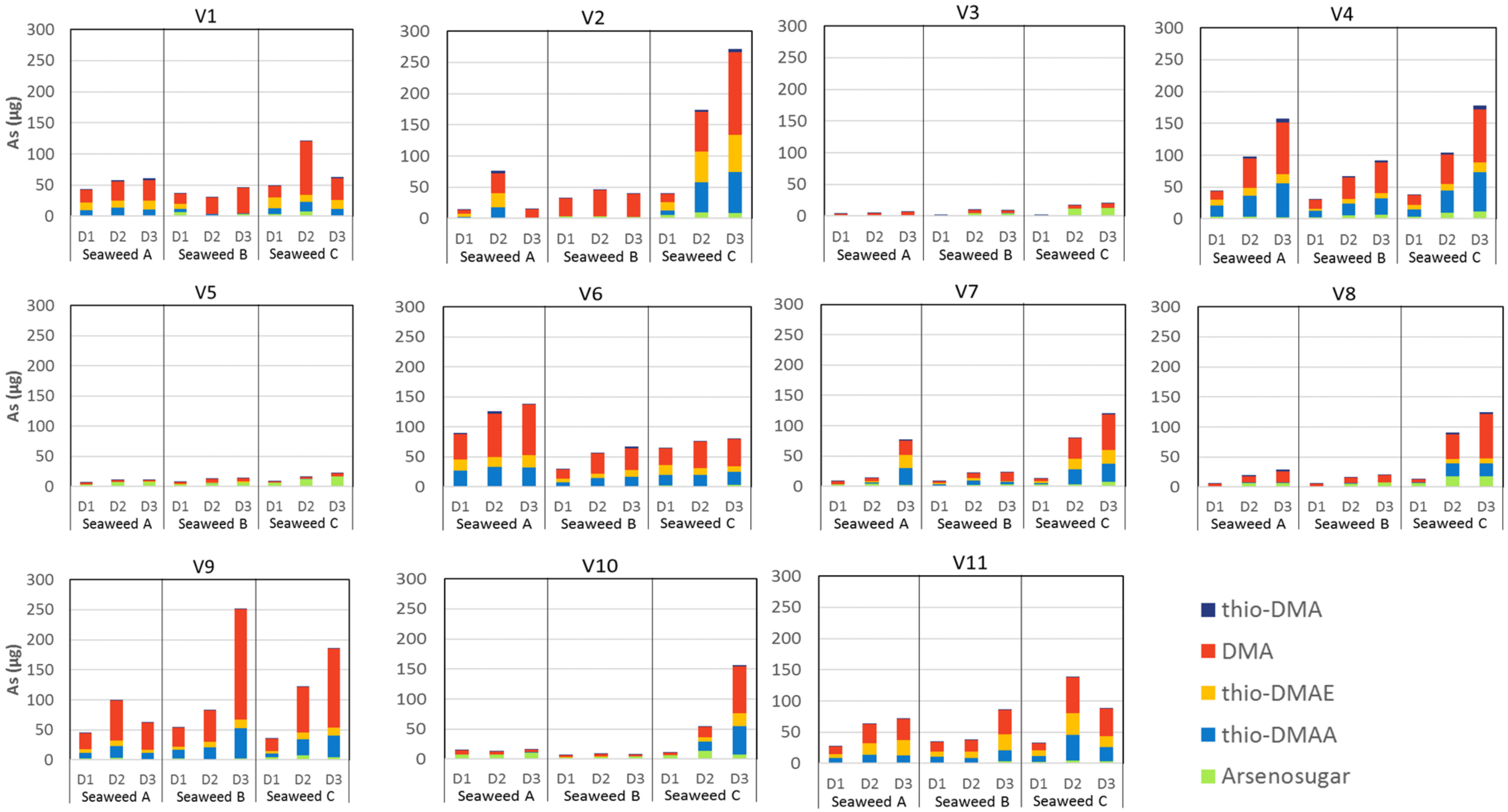

Supplement: Supplementary file 1 — Supplementary materials [file 41598_2017_3883_MOESM1_ESM.pdf]
